# Albumin Might Attenuate Bacteria-Induced Damage on Kupffer Cells for Patients with Chronic Liver Disease

**DOI:** 10.3390/cells10092298

**Published:** 2021-09-03

**Authors:** Hao Lin, Yuhui Fan, Andreas Wieser, Jiang Zhang, Ivonne Regel, Hanno Nieß, Julia Mayerle, Alexander L. Gerbes, Christian J. Steib

**Affiliations:** 1 Liver Center Munich, Department of Medicine II, University Hospital, 81377 Munich, Germany; Hao.Lin@med.uni-muenchen.de (H.L.); fyhmed@yeah.net (Y.F.); Ivonne.Regel@med.uni-muenchen.de (I.R.); Julia.Mayerle@med.uni-muenchen.de (J.M.); Gerbes@med.uni-muenchen.de (A.L.G.); 2 Max von Pettenkofer Institute, Faculty of Medicine, Medical Microbiology and Hospital Epidemiology, 80336 Munich, Germany; wieser@mvp.lmu.de; 3 Division of Infectious Diseases and Tropical Medicine, University Hospital, 80802 Munich, Germany; 4 German Center for Infection Research (DZIF), Partner Site Munich, 80802 Munich, Germany; 5 Liver Transplantation Center, Department of Liver Surgery, Ren Ji Hospital, School of Medicine, Shanghai Jiao Tong University, Shanghai 200127, China; zhangjiang@renji.com; 6 Biobank of the Department of General, Visceral and Transplant Surgery, University Hospital, 80802 Munich, Germany; Hanno.Niess@med.uni-muenchen.de

**Keywords:** hepatic non-parenchymal cells, albumin, chronic liver diseases, bacteria

## Abstract

Chronic liver diseases (CLDs) are complex diseases that cause long-term inflammation and infection, which in turn accelerate their development. The usage of albumin in patients with CLDs has been debated for years. Human serum albumin (HSA) plays a key role in immunomodulation during the process of CLDs. The correlation between albumin and C-reactive protein (CRP) in CLD patients was analyzed by linear regression with the Pearson statistic. The damage of THP-1 and primary cells was evaluated by measuring the lactate dehydrogenase (LDH) in the supernatant. Immunofluorescence staining was performed to determine underlying pathways in Kupffer cells (KCs). Albumin negatively correlated with infection in patients with CLDs. In vitro experiments with THP-1 cells and KCs showed that albumin reduced LDH release after stimulation with bacterial products, while no differences in hepatic stellate cells (HSCs) and sinusoidal endothelial cells (SECs) were detected. Moreover, immunofluorescence staining revealed an increase of p-ERK and p-NF-kB p65 density after albumin treatment of KCs stimulated by bacterial products. In conclusion, albumin could assist CLD patients in alleviating inflammation caused by bacterial products and might be beneficial to patients with CLDs by securing KCs from bacteria-induced damage, providing a compelling rationale for albumin therapy in patients with CLDs.

## 1. Introduction

Chronic liver diseases (CLDs) are a long-term pathological process involving continuous destruction and regeneration of liver parenchyma that leads to cirrhosis at its most advanced stage. The mechanism of the progression of CLDs is complicated and remains unsettled, but unceasing inflammation and bacterial infections may play an important role in this process [1,2]. Spontaneous bacterial peritonitis (SBP) is one of the most common infections during CLDs [3]. Gram-negative aerobic or facultative aerobic organisms such as *Klebsiella pneumoniae* (*K. pneumoniae*), *Escherichia coli* (*E. coli*), *Pseudomonas aeruginosa* (*P. aeruginosa*) and *Enterobacter cloacae* (*E. cloacae*) are the most common cause of SBP patients; other common organisms include the Gram-positive species *Enterococcus faecium* (*E. faecium*), *Streptococcus pneumoniae* (*S. peneumoniae*) and *Staphylococcus aureus* (*S. aureus*) [4].

Kupffer cells (KCs) are resident liver macrophages as well as the largest population of innate immune cells in the liver [5]. The major function of KCs is to scavenge and phagocyte cell debris, small particles, protein complexes and senescent red blood cells through an interplay with pattern recognition receptors. In addition, gut-derived toxic materials including pathogens from the intestinal flora and endotoxic lipopolysaccharide (LPS) are removed by KCs [5,6,7]. Thus, KCs play an essential role in maintaining the homeostasis to protect the host and in prompting immunogenic and tolerogenic immune responses.

Human serum albumin (HSA) is a crucial plasma protein often administered in the therapies of patients with CLDs. However, there still are debates on the benefit of albumin in patients with cirrhosis over the long term. Notably, a recent clinical trial presented that the long-term albumin administration to patients with cirrhosis reduced systemic inflammation [8] and the cumulative incidence of complications of cirrhosis, including SBP and non-SBP bacterial infections [9,10]. Among the reasons of benefits of albumin in patients with cirrhosis, expander function depending on its known oncotic properties is well known, although it is now irrefutably clear that the immunomodulatory function of albumin turns to be more important during the therapies for liver cirrhosis [11,12,13]. HSA is capable of binding inflammatory factors and mediators including LPS, lipoteichoic acid and peptidoglycan, which are the surface components of Gram-negative and Gram-positive bacteria capable of activating the innate immune system through Toll-like receptor 2/4 (TLR 2/4) and initiating inflammation [12,13,14,15]. Albumin preconditioning abrogated the LPS-mediated inflammation through the Nuclear Factor-κB (NF-κB) activation [16] and enhanced monocyte interleukin-6 (IL-6) gene expression via the extracellular signal-regulated kinase (ERK) and NF-κB pathways [17]. Albumin can modulate innate immune responses to sepsis and cirrhosis-associated prostaglandin E2-mediated immune dysfunction following albumin infusion [18]. Nevertheless, the role of albumin on KCs, the first barrier against bacteria and a vital role in bacteria-induced immune responses, has not been investigated.

Hence, our study underscores a protective role of albumin in patients with CLDs and in KCs stimulated by the bacterial products, suggesting that albumin therapies aim to not only improve plasma osmolality for patients with CLDs but benefit KCs in reducing the damage from microbial products, providing a convincing rationale for albumin application in patients with CLDs.

## 2. Materials and Method

### 2.1. Study Cohort

From July 2016 to March 2019, 138 outpatients or consecutive hospitalized patients with CLDs were obtained to be included in this study. The types of CLDs included in this study are: chronic viral hepatitis B (*n* = 26), autoimmune hepatitis (*n* = 21), chronic viral hepatitis C (*n* = 18), liver cirrhosis (*n* = 33, including 29 hepatitis-induced, 3 alcohol-induced and 1 Alagille syndrome-induced), primary sclerosing cholangitis (*n* = 8), primary biliary cholangitis (*n* = 6), steatosis hepatitis (*n* = 5), nonalcoholic steatohepatitis (NASH, *n* = 4), alcoholic liver disease (*n* = 3), cryptogenic cirrhosis (*n* = 3), cystic liver disease (*n* = 2), Budd–Chiari Syndrome (*n* = 2), hemochromatosis (*n* = 2), liver adenoma (*n* = 2), M. Wilson disease (*n* = 1), sarcoidosis (*n* = 1) and toxic liver disease (*n* = 1). The study protocol was approved by the ethics committee of the Faculty of Medicine at the Ludwig-Maximilians University (LMU) (approval number 17-756), and patients provided written informed consent. All indicators were measured by the clinical chemistry laboratory of the university hospital of LMU using an automatic analyzer (cobas^®^8000 modular analyzer series, Roche, Switzerland) and standardized operating procedures according to the manual. A score was calculated using the following formula: MELD Score = 10 × (0.957 × ln (Creatinine) + 0.378 × ln (Bilirubin) + 1.12 × ln (INR) + 0.643).

### 2.2. Human Tissue Specimens

Double-coded liver tissue samples and corresponding data used in this study were provided by the Biobank of the Department of General, Visceral and Transplant Surgery of LMU. This Biobank operates under the administration of the Human Tissue and Cell Research (HTCR) Foundation. The framework of HTCR Foundation [19], which includes obtaining written informed consent from all donors, has been approved by the ethics committee of the Faculty of Medicine at the LMU (approval number 025-12) as well as the Bavarian State Medical Association (approval number 11142) in Germany. A total of 10 human liver tissues were included in our experiments and were from patients with liver metastasis.

### 2.3. Primary Hepatic Non-Parenchymal Cells Isolation and Culture

Liver tissues were preserved in RPMI 1640 medium (Gibco, Karlsruhe, Germany) on ice immediately after surgical removal, and cells were isolated within 6 h. Nycodenz (Axis Shield, Rodelokka, Norway) gradients density centrifugation was performed to isolate primary liver KCs and hepatic stellate cells (HSCs). In brief, liver samples were cut into 1-5 mm thick slices and rinsed by phosphate-buffered saline (PBS). The tissue slices were digested with 15 mg/mL pronase (Sigma, St. Louis, USA) at 37 °C for 20 min. After two times of 80 μm and 60 μm nylon mesh filtration, the tissue solution was mixed with Nycodenz solution (16.7% for KCs and 28.7% for HSCs) and centrifuged (1400× *g*, 20 min) without the brake, dividing into three layers. We collected the middle layer and interfaces. To isolate sinusoidal endothelial cells (SECs), CD146 MicroBeads (Miltenyi Biotec, Teterow, Germany) and a magnetically activated cell sorting system were employed to filtrate the tissue digestive solution instead of Nycodenz. Cells were seeded in multiple well plates and cultured at 37 °C in 5% CO_2_ in RPMI 1640 with 10% fetal calf serum (FCS, PAN, Aidenbach, Germany) and 1% Penicillin-Streptomycin (Sigma, St. Louis, MO, USA). Detailed procedures were described previously [20,21,22].

### 2.4. Cell Culture and Treatment

The THP-1 cell line (American Type Culture Collection, reference number TIB-202™) was a gift from Prof. Peter Nelson. Cells were cultured at 37 °C in 5% CO_2_ in RPMI 1640 with 10% FCS and 1% Penicillin–Streptomycin. To differentiate THP-1 into adherent macrophages, 20 ng/mL phorbol myristate acetate (PMA) was used to treat cells in a complete medium for 48 h. The medium was changed into a serum-free medium before the stimulation of bacterial products. Cells were treated with albumin at the same time with (peri-treatment) or 24 h before (pre-treatment) the stimulation of bacterial products. The supernatant was harvested after 24 h of the microbial isolate stimulation.

### 2.5. Bacterial Products Isolation and Stimulation

Bacterial strains including *K. pneumoniae*, *E. coli* and *E. cloacae*, *P. aeruginosa*, *E. faecium*, *S. peneumoniae* and *S. aureus* were isolated from patients with SBP (different groups of patients from the study cohort). The isolates were cultured on Columbia 5% sheep blood media (Becton Dickinson, Heidelberg, Germany) at 37 °C under aeration. Bacterial colonies were taken off the solid media with caution not to include any parts of the media. The bacterial pellet was resuspended in phosphate-buffered saline (PBS pH 7.4) by pipetting up and down and vortex mixing. Cells were washed in PBS three times to remove any residual media or debris. After the last washing steps, the bacterial cell mass was resuspended in PBS buffer and sterilized using heat inactivation [23]. Bacterial inactivation was confirmed twice by plating the heat-inactivated extracts on media and cultivating them for 48 h at 37 °C. Bacterial product solutions were measured for protein content in serial dilutions (Bradford) and were subsequently divided into aliquots and stored frozen until use. The bacterial products solutions were diluted to various concentrations (1, 8, 16 μg/mL) with serum-free medium and then were used to stimulate cells at different concentrations with or without HSA.

### 2.6. Evaluation of Cell Damage

The lactate dehydrogenase (LDH) was used to assess the cell damage of THP-1 and primary cells. LDH catalyzes the reversible conversion of lactate to pyruvate with the reduction of NAD+ to NADH. Thus, the production of NADH was employed to determine the LDH activity indirectly, which was measured by the absorbance value at 365 nm [20,24,25]. 5% Triton-X-100, which is inducing cell damage, was applied as a positive control.

### 2.7. Immunofluorescence Staining

Cells were fixed with 4% paraformaldehyde for 10 min (Roth, Karlsruhe, Germany), then they were blocked in 10% donkey serum for 30 min, and were permeabilized with 0.1−0.5% Triton X-100 in PBS for 10 min [26]. Subsequently, cells were incubated with the primary antibodies (1:250 p-ERK and 1:1000 p-NF-kB p65, Cell Signaling Technology) overnight at 4 °C and fluorescent-labeled secondary antibodies at room temperature for 1 h the next day. The images were captured by immunofluorescence microscopy (Leica, Germany).

### 2.8. Statistical Analyses

Results are presented as mean ± standard deviation (SD) or median and interquartile range (IQR). Normality of data distribution was tested by Kolmogorov–Smirnov Z test (Table 1). Statistical comparisons by using the unpaired two-tailed Student’s *t*-test and Mann–Whitney U test were performed. Correlations between variables were calculated using linear regression with the Pearson statistic. A p-value less than 0.05 was considered significant. SPSS was used for data analysis.

## 3. Results

### 3.1. Albumin Negatively Correlated with Infection in Patients with CLDs

**Figure 1:**
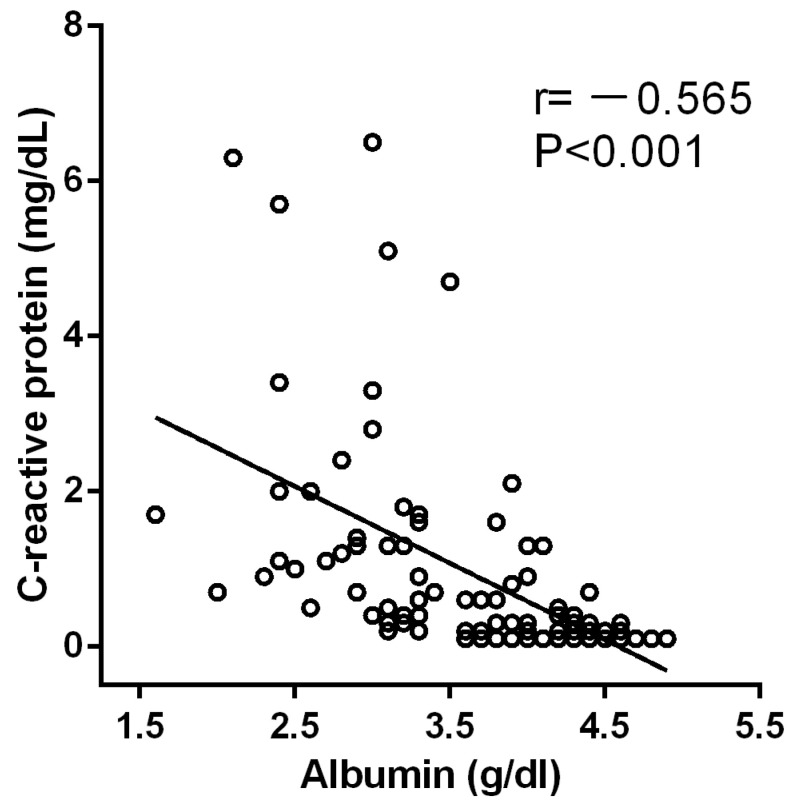
The correlation between albumin and CRP.

All 138 patients with CLDs were divided into two groups according to their HSA levels: the normal albumin group (≥3.5 g/dL) and the low albumin group (<3.5 g/dL). Table 2 displayed that CLD patients with lower albumin had higher bilirubin, aspartate aminotransferase (GOT), Gamma-GT, LDH, international normalized ratio (INR), and creatinine, C-reactive protein (CRP), MELD Score, while NaCl, KCl and Zinc serum levels were remarkably lower. Pearson correlation analysis and linear regression were performed to investigate the association between these indicators and albumin levels. As exhibited in Table 3 and Figure 1, high serum albumin levels correlated with a low concentration of CRP in patients with CLDs showing an r value of 0.565 (*p* < 0.001). Which indicated that patients with CLDs with high serum albumin had less infection. In addition, the levels of NaCl, KCl and Zinc positively correlated with albumin levels in patients with CLDs while bilirubin, GOT, Gamma-GT, LDH, INR, and creatinine had a negative correlation with albumin levels (Table 3 and Figure S1).

The CRP levels were negatively correlated with albumin levels in patients with CLDs (r = −0.565, *p* < 0.001).

### 3.2. Albumin Reduces the Cell Damage Caused by a Bacterial Infection in THP-1 Cells

**Figure 2:**
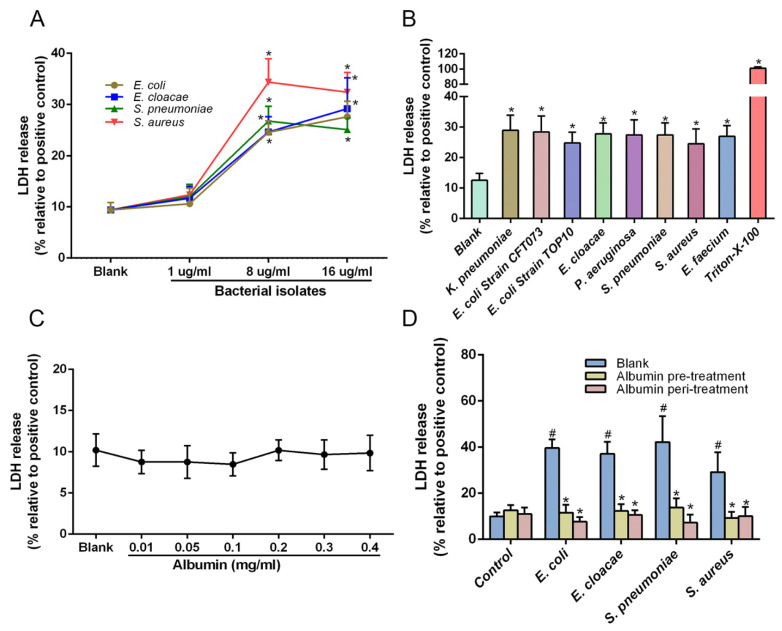
Effects of albumin on the injury induced by bacteria in THP-1. (**A**) The cell damage caused by bacteria products at various concentrations. (**B**) Measurement of the cell damage caused by bacterial components (8 μg/mL) isolated from indicated bacteria strain from patients with CLDs. (**C**) Toxic measurement of different doses of albumin in THP-1. (**D**) Effects of albumin treatment (0.4 mg/mL) on THP-1 stimulated with bacterial isolates (8 μg/mL). All LDH data are normalized to a positive control (5% Triton-X-100). The 5% Triton-X-100 was set as a positive control in all LDH measurement assays. Data expressed as means ± SD, * *p* < 0.05 vs. blank treatment in their own group, ^#^ *p* < 0.05 vs. blank treatment in the control group, *n* = 6 in each group from three independent experiments.

It is well-known that a CRP test could be used to monitor conditions that cause inflammation, including bacterial infections, fungal infections and inflammatory bowel diseases. CLD patients would most likely have SBP during the development of CLDs, and the most common reasons for SBP are bacterial invasions [3,4]. Besides this, the sensitivity and specificity of CRP are high enough to evaluate the condition of bacterial infection in patients with CLDs [27]. Together, the increase of CRP levels in CLD patients are mainly attributed to the bacterial infection in our study; therefore, we used bacterial products to stimulate THP-1 and primary cells in the subsequent experiments. Our previous publication indicated that Zinc protects KCs from microbial infection [28]. As shown in Table 3 and Figure S1, the concentration of albumin had a strong positive correlation with Zinc levels in patients with CLDs (r = 0.752, *p* < 0.001). In this study, we postulated that albumin protects macrophages from the damage of bacterial infection. Instead of KCs, we selected the macrophage cell line THP-1 to perform in vitro validation experiments, because the amounts of KCs from human liver specimens were limited. First, we optimized the concentration of bacterial products that causes LDH release, as a marker for cell damage. We treated THP-1 cells with different concentrations of bacterial products and demonstrated increased LDH levels when using 8 and 16 µg/mL of bacterial isolates (Figure 2A) Next, we tested the cell damage caused by 8 bacterial strains associated with CLDs including regular Gram-negative and Gram-positive bacteria, such as *K. pneumoniae*, *E. coli* and *E. cloacae*, *P. aeruginosa*, *E. faecium*, *S. peneumoniae* and *S. aureus*. Figure 2B showed that all of the eight most ubiquitous bacteria in CLDs can cause considerable increases in LDH levels when THP-1 cells were treated with 8 µg/mL of the bacterial products.

Moreover, we analyzed whether albumin might show toxic effects on THP-1 macrophages when treated with increasing concentrations. Notably, in a range from 0.01−0.4 mg/mL, albumin treatment of THP-1 cells did not result in a meaningful increase of LDH levels. To investigate if albumin protects macrophages from bacterial damage, we treated THP-1 cells with 8 μg/mL bacterial products of four bacteria strains including *E. coli TOP10* and *E. cloacae* (Gram-negative bacteria strains) and *S. peneumoniae* and *S. aureus* (Gram-positive bacteria strains) in combination with 0.4 mg/mL albumin. Therefore, THP-1 cells either were treated with albumin 24h before (pre-treatment) or at the same time (peri-treatment) with microbial stimulation. Our data demonstrated, consistent with our previous results, that bacterial products increased LDH release in THP-1 cells compared to the control group (^#^ *p* < 0.05, Figure 2D). Furthermore, both the pre-treatment and peri-treatment of albumin significantly decreased LDH release in four bacterial product groups compared to no-albumin (blank) treatment (* *p* < 0.05, Figure 2D). These results demonstrated that albumin protected THP-1 cells from bacteria-associated damage, irrespective of the pre-treatment or peri-treatment conditions with albumin.

### 3.3. Albumin Safeguards KCs, Rather Than HSCs and SECs from Bacteria-Induced Cell Damage

**Figure 3:**
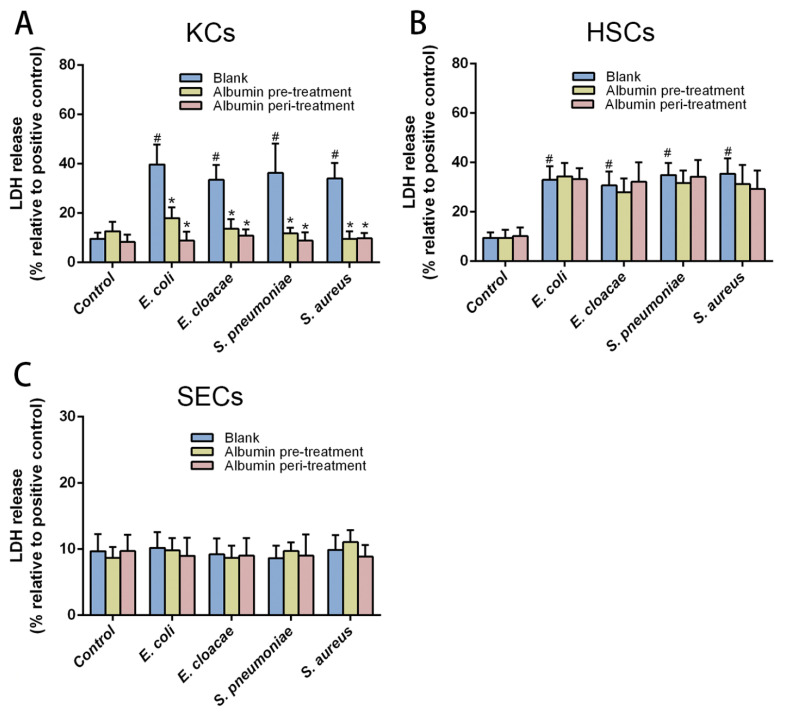
Different effects of albumin on different hepatic non-parenchymal cells. (**A**) Effects of albumin (0.4 mg/mL) on the cell damage induced by bacterial products (8 μg/mL) for KCs. No significant effects of albumin on HSCs (**B**) and SECs (**C**) were found in both pre-treatment and peri-treatment. Data expressed as means ± SD, * *p* < 0.05 vs. blank treatment in their own group, ^#^ *p* < 0.05 vs. blank treatment in the control group, *n* = 6 in each group from three independent experiments with a total of three different human livers.

To investigate whether albumin has identical effects on primary hepatic non-parenchymal cells as it was shown above for the THP-1 cell line, KCs, HSCs and SECs isolated from human liver specimens were examined. Like the THP-1 cells, primary KCs, HSCs and SECs were treated with albumin and stimulated by bacterial products. LDH was measured to assess cell damage. Albumin treatment of KCs remarkably reduced LDH release caused by bacterial isolates in both pre-and peri-treatment conditions. Interestingly, for HSCs, the bacterial products notably increased LDH levels (^#^ *p* < 0.05); however, no significant changes in LDH levels were found after albumin treatment (Figure 3B). In addition, bacterial product stimulation and albumin treatment did not induce any notable changes in LDH levels in SECs (Figure 3C). From these findings, we conclude that albumin has a protective role on KCs preventing KC damage during a bacterial invasion in CLD patients.

### 3.4. ERK and NF-kB Pathways Were Involved in the Protective Effects of Albumin

**Figure 4:**
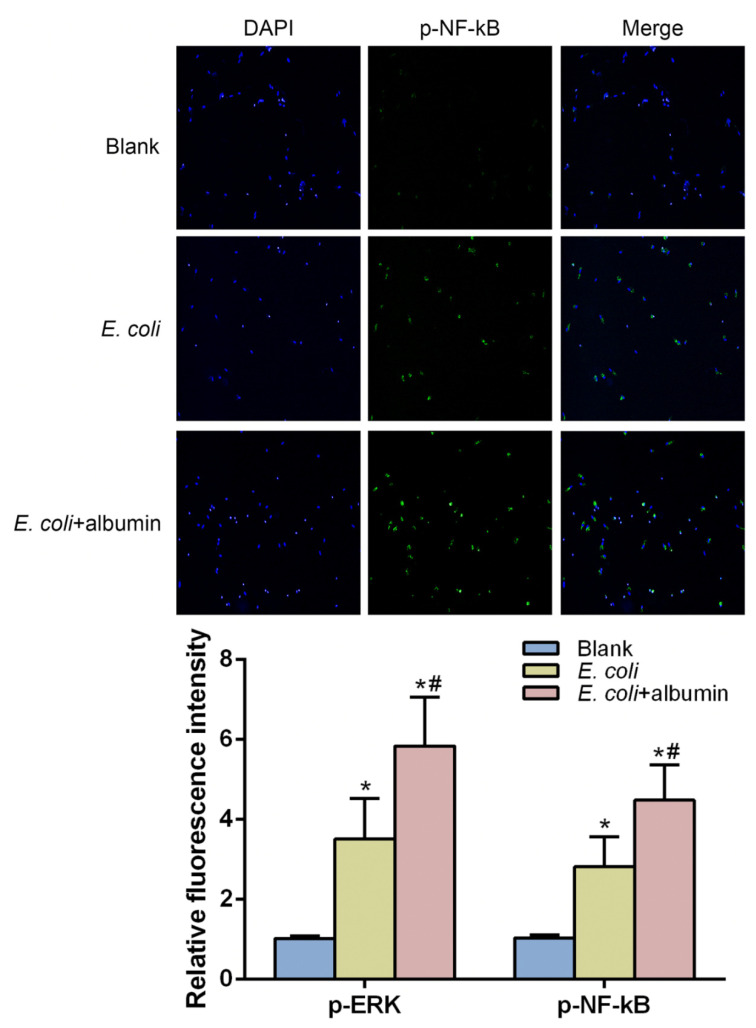
The potential mechanism of the protective effects of albumin in KCs. Immunofluorescence labeling of p-ERK and p-NF-kB protein (green) of KCs treated with albumin and *E. coli*. The nuclei were stained with 4′-6-diamidino-2-phenylindole (blue). Scale bars, 100μm. The relative fluorescence intensity compared with DAPI intensity in the staining experiments was measured. Data expressed as means ± SD, * *p* < 0.05 vs. blank group, ^#^ *p* < 0.05 vs. *E. coli* group, *n* = 6 in each group from three independent experiments with a total of three different human livers.

ERK and NF-kB pathways are the most important pathways participating in the TLR/MyD88/IRAK4 axis that is activated by Gram-positive and Gram-negative bacteria in livers [29,30,31]. To determine the activation of the ERK and NF-kB pathway in KCs, we performed immunofluorescence staining of phosphor-ERK (p-ERK) and phosphor-NF-kB (p-NF-kB) under control, *E. coli* and *E. coli* plus albumin treatment. The results showed that albumin intensifies the staining density of p-ERK and p-NF-kB compared to the control group and *E. coli* group in KCs (Figure 4). These findings provided preliminary evidence of the involvement of ERK and the NF-kB pathway in the process of albumin protection on liver macrophages.

## 4. Discussion

In the present study, we described for the first time the beneficial effects of albumin reducing the damage of KCs caused by bacteria infections. Albumin is applied regularly to improve osmotic pressure for patients with cirrhosis, but there is only little information on further functions of albumin. This study highlighted the relationship of albumin and inflammation after bacteria invasion in patients with CLDs, the immune-related role of albumin in patients with CLDs, and its underlying mechanism. Novel findings in this study were: (1) In patients with CLDs, albumin levels were found to be inversely correlated to CRP levels, a marker for infection. (2) Albumin protects KCs against bacterial-induced cell injury, but no effects were observed in HSCs. (3) The ERK and NF-kB pathway may be involved in the protective effects of albumin treatment in KCs.

In patients with CLDs, the clinical benefits of albumin therapy have been debated for years. Several reports have failed to show any favorable effect on the administration of albumin in CLDs except for delaying the onset of renal failure [32,33]. Nevertheless, the wide range of potential benefits of albumin administration, such as anti-inflammatory activity, antioxidant function, immunomodulation and the transportation of many endogenous and exogenous substances, still need to be further investigated [34]. Recently, there were some important clinical trials conducted to supply more convincing evidence of the benefits of albumin administration in patients with CLDs. The data indicated that long-term albumin supplementation to patients with cirrhosis and ascites improved survival, lowered hospitalizations and prevented complications [8,9,35]. In our present study, we found that patients with lower albumin levels had more severe inflammation (Figure 1), suggesting that albumin eased the inflammation in patients with CLDs and participated in decreasing the immune responses due to the bacterial invasion. Albumin has the ability to bind and inactivate many inflammatory mediators, such as pathogen-associated molecular patterns, reactive oxygen species, bioactive lipid metabolites and nitric oxide, which could be the underlying reason for improving the inflammation for patients with CLDs with normal albumin.

The immunomodulatory function of albumin has been increasingly studied recently. Decreased serum albumin in patients with cirrhosis binds fewer prostaglandin E2 (PGE2) resulting in increased bioavailability of PGE2, which dampens the macrophage response to LPS [18], indicating that albumin may promote immune functions in some ways. KCs are one of the most important immune cells participating in the immune reaction. To explore the relative mechanism of albumin on reducing the inflammation presented in Figure 1, we treated KCs with albumin in our study. Another study showed no positive changes under the administration of albumin on patients with cirrhosis [36], even though there were three clinical trials to prove the benefit of albumin on patients with CLDs referred above. Interestingly, a loading dose of albumin administration in the other three studies has been considered to be the reason behind this difference [12], which means that the concentration of albumin administration is critical to show its advantages. In addition, it has previously been observed that 0.4 mg/mL albumin could improve PGE2-mediated immunosuppression [18]. Taken together, 0.4 mg/mL albumin was used in our cell experiments. KC isolation from the human liver is a tricky technique, and the number of cells was limited. THP-1 is a human monocytic cell line obtained from a patient with acute monocytic leukemia, and the cells differentiated with PMA are commonly utilized as a model for human macrophage function and biology [36]. Therefore, we used them rather than KCs to measure the damage from different strains and various concentrations of bacterial products. Our results presented that 8 μg/mL and 16 μg/mL of bacterial product stimulation had a significant injury on THP-1 cells (Figure 2A). Bacterial products of all tested strains damaged THP-1 cells regardless of Gram-negative or Gram-positive bacteria (Figure 2B), whereas albumin with increasing concentrations had no toxic effects on cells (Figure 2C).

Next, we treated KCs and THP-1 with albumin to examine its protective effects on primary macrophages. Our data showed that albumin treatment before and during the stimulation with bacterial products protected KCs and THP-1 from injury by microbial isolates (Figure 2D and Figure 3A), which might be caused by the capability of albumin to bind inflammatory factors as mentioned above. Another possible explanation for this is that the albumin might strengthen the phagocytosis of KCs, and increase the albumin levels inside cells, and this might have a synergistic effect on the protection of KCs. Besides, we confirmed our previous conclusion that microbial isolates did damage to KCs and HSCs rather than SECs (Figure 3B,C) [28]. However, one unanticipated finding was that albumin treatment had no notable protective effects on HSCs (Figure 3B). The receptor of polymerized albumin was found on macrophages [37] and its role for endocytosis of albumin has been described [38]. Similarly, stellate cells can actively take up albumin from extracellular sources [39]. Nonetheless, the phagocytosis of macrophages is stronger than stellate cells, which might be a possible mechanism behind this unexpected result.

Taken together, we initially had speculated that patients with CLDs with normal albumin could inactive inflammatory factors and bacterial products. Furthermore, it allows KCs to function normally and facilitate an immune response to foreign bacteria. We hypothesized that at the beginning of immune responses, CLD patients with normal albumin might have more intense inflammation and higher CRP for a short time (acute inflammation responses) due to the response of a healthy immune system, then finally return to normal with the development of the effective immune responses maintained by the protective effect of albumin on KCs, which can explain the relationship between our CRP data in patients with CLDs in Figure 1 and the cell damage assay in Figure 2D and Figure 3A. Thus, we conjectured that the protective effect of albumin on KCs might confer some benefits of diminishing the inflammation in patients with CLDs.

Our previous publication revealed that Zinc protects KCs from this damage caused by bacterial isolates [28]. Considering similar protective effects of Zinc and albumin, we hypothesized that a potential interacting mechanism of these effects might exist. Bacteria or bacterial products stimulate TLRs which are the essential molecules involved in bacteria-induced inflammation in macrophages. Activation of TLRs triggers ERK and NF-κB signaling pathways, which are critical for a normal immune response [29,30]. A previous study demonstrated that albumin treatment produces a dose-dependent increase in pro-inflammatory gene expression (acute inflammation responses) in vitro through activation of ERK and NF-κB pathways [40,41,42,43]. Thus, we speculated that the protective effects of albumin on liver macrophages are dependent on the ERK and NF-kB pathways. An amplifying effect of albumin on the activation of the ERK and NF-kB pathways caused by bacteria products was observed compared to the *E. coli* stimulation (Figure 4). Together, our findings provided possible mechanisms for the effect of albumin and aided in understanding the therapeutic benefits of albumin on patients with CLDs.

**Figure 5:**
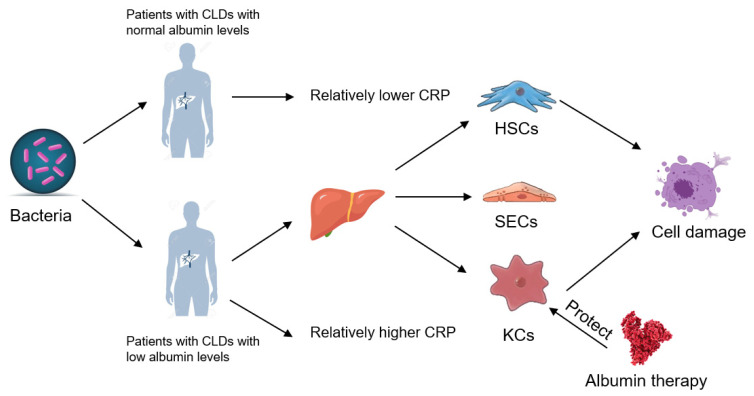
A proposed role of albumin in patients with CLDs and different hepatic non-parenchymal cells.

In conclusion, our results revealed that albumin might help patients with CLDs to better overcome the effects caused by bacterial infection and defend KCs from the injury caused by bacteria in patients with CLDs (Figure 5), implying immunomodulatory-related benefits of albumin administration on patients with CLDs. Thus, we conjectured that albumin supplementation might mitigate bacterial infection in patients with CLDs.

Bacteria raised higher CRP levels in patients with CLDs with low albumin than CLD patients with high albumin. HSCs and SECs instead of SECs could be demolished by bacteria. However, albumin could protect KCs rather than HSCs from the cell damage caused by bacteria.

## Figures and Tables

**Table 1:** Normality test of laboratory parameters.

Characteristic	All (N)	K-S Test	*p*-Value
Age (years)	53.45 + 14.66 (138)	0.642	0.804
NaCl (mmol/L)	137.8 + 3.63 (138)	1.699	0.006
KCl (mmol/L)	4.3 + 0.47 (138)	1.079	0.195
Zinc (mmol/L)	67.83 + 20.87 (138)	0.782	0.573
CRP (mg/dL)	0.98 + 1.86 (106)	3.177	<0.001
Leukocytes (G/L)	6.38 + 2.34 (137)	1.191	0.117
Bilirubin (mg/dL)	2.55 + 4.71 (138)	3.717	<0.001
GOT (U/L)	116.19 + 426.89 (132)	4.757	<0.001
GPT (U/L)	154.49 + 673.88 (138)	5.071	<0.001
Gamma-GT (U/L)	106.31 + 130.97 (138)	2.699	<0.001
LDH (U/L)	247.17 + 160.85 (71)	2.226	<0.001
INR	2.22 + 0.39 (138)	2.595	<0.001
Creatinine (mg/dL)	1.07 + 0.86 (138)	3.635	<0.001
MELD Score	10.79 + 6.7 (138)	2.787	<0.001

K-S test: Kolmogorov–Smirnov Z test. Data are expressed as mean ± SD.

**Table 2:** Characteristic analysis based on different albumin concentrations.

Characteristic	Normal Albumin (n)	Low Albumin (n)	*p*-Value
	*N* = 99	*N* = 39	
Age (years)	52.92 + 15.49 (99)	54.80 + 12.40 (39)	0.501 ^a^
Sex (Male/Female)	42/57	23/16	/
NaCl (mmol/L)	138(139–140) (99)	135(133–138) (39)	<0.001 ^b^
KCl (mmol/L)	4.45 + 0.38 (99)	3.40 + 0.43 (39)	<0.001 ^a^
Zinc (mmol/L)	75.96 + 17.14 (99)	47.15 + 14.15 (39)	<0.001 ^a^
CRP (mg/dL)	0.10(0.10–0.30) (67)	1.30(0.60–2.00) (39)	<0.001 ^b^
Leukocytes (G/L)	6.54 ± 2.24 (98)	5.97 + 2.55 (39)	0.195 ^a^
Bilirubin (mg/dL)	0.70(0.50–1.10) (99)	2.9(2.3–10.5) (39)	<0.001 ^b^
GOT (U/L)	29.00(25.00–39.00) (99)	73.00(42.00–118.00) (35)	<0.001 ^b^
GPT (U/L)	30.00(21.00–49.00) (99)	33.00(19.00–93.00) (39)	0.192 ^b^
Gamma-GT (U/L)	37.00(22.00–98.00) (98)	147.00(55.00–202.00) (39)	<0.001 ^b^
LDH (U/L)	197.50(166.50–234.75)	238.00(195.50–316.00) (28)	0.003 ^b^
INR	1.00(0.90–1.10) (99)	1.40(1.20–1.60) (39)	<0.001 ^b^
Creatinine (mg/dL)	0.80(0.90 + 1.00) (99)	1.20(0.90–1.40) (39)	<0.001 ^b^
MELD Score	7.00(6.00–8.00) (99)	17.00(13.00–22.00) (39)	<0.001 ^b^

Data are expressed as mean ± SD or median and interquartile range (IQR); ^a^ Student’s *t*-test.; ^b^ Mann–Whitney U test.

**Table 3:** Correlation between characteristic and different albumin concentrations.

Characteristic	Normal Albumin		Low Albumin		All	
	R	*p*-Value	R	*p*-Value	R	*p*-Value
Age (years)	−0.358	<0.001	−0.0101	0.541	−0.189	0.026
NaCl (mmol/L)	0.084	0.408	0.635	<0.001	0.637	<0.001
KCl (mmol/L)	0.145	0.152	0.037	0.823	0.504	<0.001
Zinc (mmol/L)	0.466	<0.001	0.460	0.003	0.716	<0.001
CRP (mg/dL)	−0.409	0.001	−0.308	0.056	0.565	<0.001
Leukocytes (G/L)	0.090	0.376	−0.238	0.145	0.092	0.286
Bilirubin (mg/dL)	−0.273	0.006	−0.067	0.686	−0.495	<0.001
GOT (U/L)	−0.217	0.032	0.132	0.450	−0.221	0.011
GPT (U/L)	−0.115	0.258	0.102	0.538	−0.077	0.375
Gamma-GT (U/L)	−0.299	0.003	0.102	0.537	−0.344	<0.001
LDH (U/L)	−0.128	0.43	−0.103	0.949	−0.316	0.009
INR	−0.204	0.043	−0.395	0.013	−0.604	<0.001
Creatinine (mg/dL)	−0.42	0.68	−0.080	0.627	−0.293	<0.001
MELD Score	−0.337	0.001	−0.227	0.165	−0.022	0.828

R and *p* values were obtained using the Pearson correlation test.

## Data Availability

The data presented in this study are available on request from the corresponding author.

## Supplementary Materials

The following are available online at https://www.mdpi.com/article/10.3390/cells10092298/s1, Figure S1: Correlation between albumin and various clinical characteristics.
