# Supplementary material for: Albumin Might Attenuate Bacteria-Induced Damage on Kupffer Cells for Patients with Chronic Liver Disease
Source: Cells. 2021 Sep 3;10(9):2298. doi: 10.3390/cells10092298 (PMC8469739; doi:10.3390/cells10092298)
Supplement: Supplementary file 1 [file cells-10-02298-s001.zip › cells-1346696-supplementary.pdf]

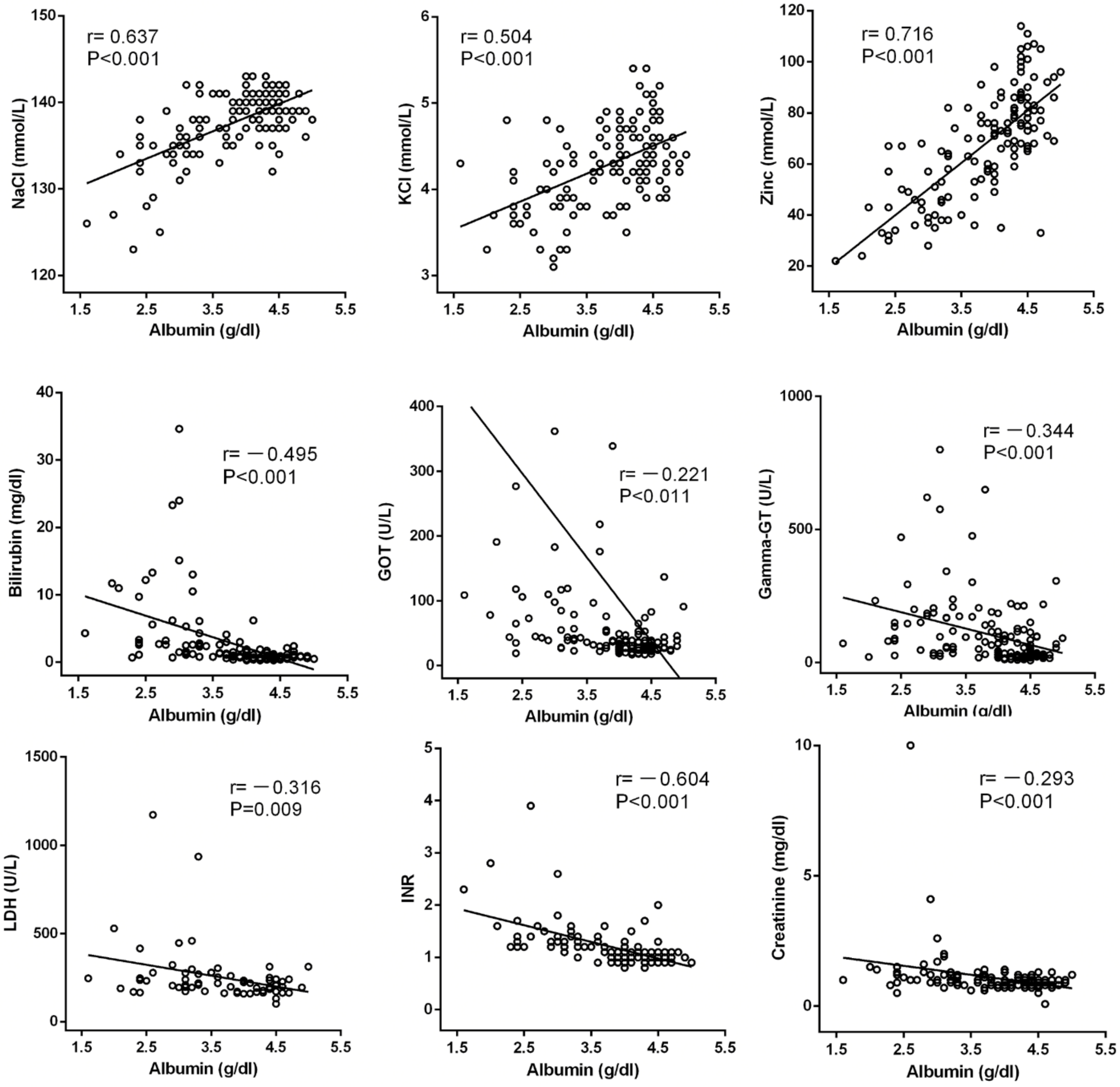

Figure S1: Correlation between albumin and various clinical characteristics. The NaCl, KCl, and Zinc levels positively correlated with albumin levels while bilirubin, GOT, Gamma-GT, LDH, INR, and creatinine negatively correlated with albumin levels in patients with CLDs.
